# Impact of legumes and plant proteins consumption on cognitive performances in the elderly

**DOI:** 10.1186/s12967-017-1209-5

**Published:** 2017-05-22

**Authors:** Elisa Mazza, Antonietta Fava, Yvelise Ferro, Marta Moraca, Stefania Rotundo, Carmela Colica, Francesco Provenzano, Rosa Terracciano, Marta Greco, Daniela Foti, Elio Gulletta, Diego Russo, Domenico Bosco, Arturo Pujia, Tiziana Montalcini

**Affiliations:** 1 Department of Clinical and Experimental Medicine, Nutrition Unit, University Magna Grecia, Viale S. Venuta, 88100 Catanzaro, Italy; 2 Department of Medical and Surgical Science, Nutrition Unit, University Magna Grecia, Catanzaro, Italy; 3 Department of Pharmacology, CNR, ISN, University Magna Graecia, Catanzaro, Italy; 4 Department of Health Science, Laboratory Unit, University Magna Grecia, Catanzaro, Italy; 5 Neurology Unit, S. Giovanni di Dio Hospital, Crotone, Italy

**Keywords:** Principal Components Analysis, Plant protein, Legumes, Cognitive decline, Elderly, Mediterranean diet

## Abstract

**Background:**

Numerous studies have investigated the role of the dietary factors in the prevention of cognitive decline but the short-term effects of foods choice on cognitive performances in the elderly are poorly explored. Our aim was to investigate the choice of foods among elderly Italian individuals and the association with cognitive function.

**Methods:**

In this longitudinal study, the participants were 214 individuals aged ≥65 years with a score >20 at the Mini Mental State Examination. The cognitive sub-test of ADAScale was used to detect cognitive decline progression over 12 months. Food choices was measured by a combination of a 24-h recall and a seven-day diet record and Principal Components Analysis.

**Results:**

The Principal Components Analysis identified four food and four nutrient patterns. MMSE and ADAS-cog score after 1 year were found to be associated with legumes pattern (B = 0.25, p = 0.007; 95% CI 0.07/0.44; and B = −0.10, p = 0.006; CI −0.79/−0.30, respectively). A dietary pattern including plant proteins was independently associated with an improved ADAS-cog after 1 year (B = 0.584, p = 0.04; OR 1.79, CI 0.04–0.42).

**Conclusions:**

The Principal Components Analysis is useful to investigate the choice of foods and nutrients in the elderly. We demonstrated an association between legumes pattern with cognitive performances.

## Background

Cognitive decline is a complex process that is affected by both genetic and environmental factors. Research over the past fifteen years have provided strong evidence for the influence of dietary factors on specific mechanisms that maintain mental function [1–5]. In this regard, it is well known that a high adherence to a Mediterranean diet (MeDi) is associated with a reduced risk of developing cardiovascular risk factors which, in turn, may be associated with the development of cognitive impairments [2, 3]. Intake of fish and vegetables and nutrients such as vitamins and minerals since a young age or adulthood may lower the risk of cognitive impairment [6]. However, there is still considerable scientific uncertainty about the relationship between a healthy diet and the risk of Dementia [7]. Furthermore, the short-term effects of foods choice in the elderly are poorly explored. Eating can be difficult for elderly because of the numerous changes that occur with age and eventual presence of diseases [8]. Elderly patients also present with sensory losses due to age which may be responsible for a lack of interest in several foods [8]. Thus, we performed a study with the aim to analyse the choices and consumption of foods among elderly Italian individuals and to investigated the association between their dietary patterns and cognitive function.

## Methods

This is a longitudinal study conducted from February 2013 to December 2014, whose protocol was approved by the local ethics committee at the “Mater Domini” University Hospital in Catanzaro, Italy (Projects Codes 2011.48).

A total of 214 community-dwelling, white individuals aged ≥65 years underwent a neuropsychological assessment conducted by expert neurologists using a medical assessment and two neuropsychological tests, which were the following: the Mini Mental State Examination (MMSE) and the Alzheimer’s Disease Assessment Scale-Cognitive sub-scale (ADAS-cog) [9–16].

The participants were from a Mediterranean area (Calabria region, southern Italy) and, as in other investigations of the elderly, were invited to participate in the study by newspapers advertisements [17, 18]. All subjects had an MMSE score greater than 20 [10–13], were literate and were not suffering from any debilitating diseases (like stage 2–5 chronic kidney disease, end stage liver failure, cancer, congestive heart failure). They had no previous history of cardiovascular disease (CVD) or thyroid dysfunction or excessive alcohol consumption and did not take any dietary supplements, psychotropic drugs as ascertained from their medical history, a physical and neurological examination and laboratory tests.

Since it has been already demonstrated that the ADAS-Cog can be the main outcome measure also in cognitively normal individuals [9], we performed a longitudinal evaluation lasting 12 months with the ADAS-Cog as the main outcome. This part of the study involved only a sample consisting of the first 144 subjects who had complete data on ADAS-Cog scores at the follow-up visit.

### Neuropsychological assessment

The neuropsychological assessment was conducted by a medical assessment alongside the use of the MMSE and ADAS-cog.

MMSE is a global test of cognitive function with components of orientation, attention, calculation, language and recall [10]. A score of 20 or below is indicative of cognitive impairment [11, 12]. A validated Italian version was used [14].

The ADAS-cog is a psychometric scale, measuring memory disturbances, language, praxis, attention and other cognitive abilities [15]. The range of scores is from 0 to 70 and the scale of the ADAS-cog is reversed, where 0 represents no errors and 70 represents errors on all items [15, 16].

To reduce the potential for practice effects during subsequent visits, different word lists in the neuropsychological tests were used. In addition, the investigators performing the cognitive tests were blinded to the patients’ clinical data and nutrient intake.

In all patients we assessed the presence of the known classical cardiovascular (CV) risk factors and anthropometric characteristics. The following criteria were used to define the distinct CV risk factors: diabetes: fasting blood glucose ≥126 mg/dL or antidiabetic treatment; hyperlipidemia: total cholesterol >200 mg/dL and/or triglycerides >200 mg/dL or lipid lowering drugs use; hypertension: systolic blood pressure ≥130 mmHg and/or diastolic blood pressure ≥85 mmHg or antihypertensive treatment; overweight: 25 kg/m^2^ ≤ BMI < 30 kg/m^2^; obesity: body mass index (BMI) ≥30 kg/m^2^; smoking: current smoker: who has smoked more than 100 cigarettes in their lifetime and smoke cigarettes every day or some days [19, 20]. Written informed consent was obtained from participants. The investigation conforms to the principles outlined in the Declaration of Helsinki [21].

### Dietary intake assessment

Dietary intake data were assessed by a 24-h recall and a seven-day diet record and calculated using nutritional software MetaDieta 3.0.1 (Metedasrl, San Benedetto del Tronto, Italy). The 24-h recall was collected via an interview by a dietitian who used images associated with a comprehensive food list in the program. All participants were also given a food diary, measuring sheet with life-size images of a spoon, cup and bottle sizes for food diaries. The INRAN (National Institute of Food Research) 2000 and IEO (European Institute of Oncology) 2008 database serves as the source of food composition information in the program [22]. We focused on major food groups, like consumption of fruits fresh and dry, virgin olive oil, legumes, fish, cereals, red meat, animal fats/margarines and cake/pies. The resulting database was exported into SPSS for analysis [23, 24].

### Blood pressure measurement

The measurement of the systemic blood pressure (systolic blood pressure—SBP and diastolic blood pressure—DBP) of both arms was obtained by auscultatory blood pressure technique with aneroid sphygmomanometer. Clinic BP was obtained in supine patients, after 5 min of quiet rest as previously described [25].

### Biochemical evaluation

Venous blood was collected after fasting overnight into vacutainer tubes (Becton & Dickinson, Plymouth, England) and centrifuged within 4 h. Serum glucose, total cholesterol, high density lipoprotein (HDL)-cholesterol, triglycerides and creatinine were measured with Enzymatic colorimetric test. Low-density lipoprotein (LDL) cholesterol level was calculated by the Friedewald formula [26]. Quality control was assessed daily for all determinations.

### Anthropometric measurements

All tests were performed after a 12 h overnight fast. Body weight was measured before breakfast with the subjects lightly dressed, subtracting the weight of clothes. Body weight was measured with a calibrated scale and height measured with a wall-mounted stadiometer. BMI was calculated with the following equation: weight (kg)/height (m)^2^. Waist circumferences and hip circumferences (WC and HC) were measured with a nonstretchable tape over the unclothed abdomen at the narrowest point between costal margin and iliac crest and over light clothing at the level of the widest diameter around the buttocks, respectively, as described in the past [27].

### 12 months follow-up

Dietary guidance was provided to all enrolled patients to promote a “healthy diet” by an experienced dietitian, not including a total energy reduction or weight loss goals, nor was physical activity promoted for the entire duration of the study. The enrolled patients were contacted again after 6 months by way of telephone, reminding them to maintain their dietary pattern. In those with nutrient deficiencies or severe obesity, additional assistance was provided so as to achieve their correct nutrient intake. However, these were excluded from the follow-up study.

### Data analysis

Data are reported as mean ± SD. To find a correlation between ADAS-cog (basal and follow-up) and a specific food or dietary pattern with an r equal to 0.15–0.20 and 80% power on a two-sided level of significance of 0.05, 190 subjects are required. Changes in MMSE and ADAS cog score from baseline to follow-up were compared using paired Student’s *t* test (two tailed).Significant differences were assumed to be present at *p* < 0.05 (two-tailed). All comparisons were performed using SPSS 17.0 for Windows (IBM Corporation, New York, NY, USA).

### Food pattern analysis

We obtained food patterns by using the Principal Components Analysis (PCA) [28]. We also obtained, for the first time, “nutrient patterns”, using macronutrients and micronutrients as variables in place of foods groups. The food/nutrient variables were correlated together (correlation coefficients *r* > 0.4). The orthogonal rotation (obtained with varimax option) was used to derive food patterns (non correlated components). To fix the number of components to retain, the two screen plots of the eigenvalues, that derived from the correlation matrix of the standardized variables, were examined. The eigenvalue is a value that indicates the proportion of the variance in consumption explained by each component. According to the Kaiser criterion, the number of components that should be retained from principal components analysis is equal to the number of eigenvalues that are greater than one. The food/nutrients patterns were named according to scores of the foods/nutrients that correlated most with the factor (>0.4). A higher absolute values in the correlation coefficients indicated that the food/nutrient variable contributes most to the construction of the component. Participants were globally grouped into four different classes according to food patterns and four different according to nutrient patterns (components). The Pearson’s correlation was performed to identify the variables which correlated with the patterns above cited. We analyzed the correlation with the following variables: MMSE (basal and at follow-up), ADAS-cog (basal and at follow-up), age, education level, BMI, WC, glucose, LDL, HDL, triglycerides, SBP, DBP. Furthermore, a stepwise multivariable linear regression analysis was used to test the association between MMSE and ADAS-cog (after 1 year) and each dietary pattern, if this last was in correlation with these neuropsychological tests in the univariate analysis. Finally, we performed a logistic regression analysis to assess which pattern was correlated with an improvement on ADAS-cog test after 1 year.

## Results

### Baseline characteristics and factors associated with MMSE and ADAS-cog score

Table 1 shows the characteristics of the study population. The mean age was 70 ± 4 years, the MMSE score was 24 ± 1 and ADAS-cog was 16 ± 7. The participants’ nutritional intake and food groups used in the PCA are showed in Table 2. Globally, plant-based protein sources represented 37.5% of the total protein consumed.

**Table 1:** Demographic and clinical characteristics of the whole population

Variables	Mean	SD
Age (years)	70	4
Education level (years)	11	5
BMI (kg/m^2^)	28	4
WC (cm)	96	11
HC (cm)	104	8
SBP (mmHg)	133	16
DBP (mmHg)	80	9
Glucose (mmol/L)	5.7	1.3
HDL-cholesterol (mmol/L)	1.5	0.4
LDL-cholesterol (mmol/L)	3.2	0.9
Triglycerides (mmol/L)	1.3	0.6
Creatinine (µmol/L)	72.2	17
Neuropsychological assessment		
MMSE	24	1
ADAS-Cog	16	7
Prevalence		
Smokers (%)	10	
Hyperlipidemia (%)	44	
Lipid-lowering agents	34	
Hypertension (%)	72	
Antihypertensive agents (%)	67	
Diabetes/carbohydrate intolerance(%)	20	
Oral hypoglycemic agents (%)	12	

*BMI* body mass index, *WC* waist circumference, *HC* hip circumference, *SBP* systolic blood pressure, *DBP* diastolic blood pressure, *HDL* high density lipoprotein, *LDL* low density lipoprotein, *MMSE* mini mental state examination, *ADAS-Cog* Alzheimer’s disease assessment scale-cognitive sub-scale

**Table 2:** Characteristics of the whole population: nutrient and food groups assessment

Variables	Mean	SD
Calories intake (kcal)	1916	467
Alcohol (g)^a^	4	6
Carbohydrates (g)^a^	116	17
Fats (g)^a^	38	7
Proteins (g)^a^	40	7
Animal fats (g)^a^	16	9
Plant fats (g)^a^	24	9
Animal proteins (g)^a^	26	7
Plant proteins (g)^a^	15	3
Monounsaturated fatty acids (g)^a^	20	4
Polyunsaturated fatty acids (g)^a^	4	1
Food groups		
Cereals (g)^a^	109	46
Legumes (g)^a^	10	12
Fruit (g)^a^	189	113
Fish (g)^a^	33	29
Virgin olive oil (g)^a^	19	8
Meat (g)^a^	43	26
Animal fats/margarines (g)^a^	0.4	1.8
Cakes/pies (g)^a^	20	18

^a^Adjusted for 1000/kcal

### Changes in ADAS-cog after 1 year and food patterns analysis

MMSE and ADAS-cog scores improved at the second time point (MMSE: from 24.4 ± 1 to 25.6 ± 1, p < 0.001; ADAS-cog: from 15.4 ± 5 to 13.1 ± 5, p < 0.001). Total loss at follow-up were 70 individuals, of whom 59 failed to attend to visit, 1 died, and 20 were untraceable.

**Fig. 1:**
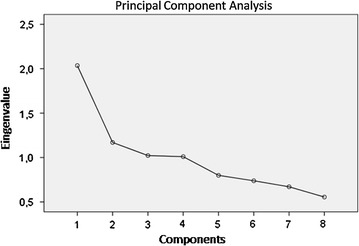
Screen plot of the eigenvalues—number of components (food patterns)

**Fig. 2:**
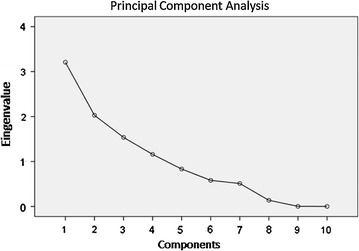
Screen plot of the eigenvalues—number of components (nutrient patterns)

Figures 1 and 2 shows the screen plot of the eigenvalues, indicating the number of components (food and nutrients patterns) obtained. In our investigation, a four-(component) food patterns, namely: (1) cereals/meat/fish/olive oil pattern, (2) cakes/fruit pattern, (3) animal fats/margarines pattern and (4) legumes pattern; solution was selected. In addition, four-nutrient patterns, namely: (1) animal protein pattern, (2) vegetal oils pattern; (3) fats pattern and (4) plant proteins/polyunsaturated fats pattern, were identified. The correlation coefficients indicated that the food or nutrient variable contributes most to the construction of the component. To simplify this presentation, only the significant data are here shown, i.e. the only patterns associated with MMSE and/or ADAS-cog score and/or improvement on ADAS-cog, that were the following:

As food patterns: (1) legumes pattern (explained variation 66%; 26 g/day = 3 serving/week); (2) animal fats/margarines pattern;

As nutrients patterns: (1) plant protein/polyunsaturated fats (variation 79%); (2) fats pattern. Table 3 shown the factors which were correlated with these patterns. In particular, legumes pattern was correlated with MMSE and ADAS-cog at basal and after 1 years (with 12-months ADAS-cog: r = −0.12 and p = 0.06; with 12-months MMSE: r = 0.21 and p = 0.01) and plant protein/polyunsaturated fats with an improved ADAS-cog test after 1 years(r = 0.18 and p = 0.03). In the stepwise multivariable linear regression analysis (Table 4), legumes pattern was found to be associated with the both ADAS-cog and MMSE after 1 year (B = −0.10, p = 0.006; 95% CI −1.79/−0.30 and B = 0.23, p = 0.01; 95% CI 0.04/0.42, respectively). Finally, vegetal proteins/polyunsatured fats pattern was found to be associated with the improved ADAS-cog (categorical variable) after 1 years (B = 0.584, p = 0.04; OR 1.79, CI 0.04–0.42, Table 5) (improved individuals were all those with a reduction of the ADAS-cog score after 1 years; not improved were those with unchanged or increased score).

**Table 3:** Univariate analyses—factors correlated with food (component 3 and 4) and nutrient (component 3 and 4) patterns

Variables		Education level	Age	ADAS-Cog improved	
Plant proteins/polyunsatured fats pattern	r	0.18	−0.14	0.18	
Nutrient component 4	p	0.008	0.035	0.030	

		WC	BMI		
Fats pattern	r	0.12	0.15		
Nutrient component 3	p	0.080	0.023		

		MMSE	MMSE	ADAS-Cog	
		Improved	12-months	Basal	12-months
Legumes pattern	r	0.15	0.21	−0.12	−0.23
Food component 4	p	0.062	0.01	0.068	0.004

		BMI			
Animal fats/margarines pattern	r	0.12			
Food component 3	p	0.06

**Table 4:** Multivariable linear regression analysis—factors associated with MMSE and ADAS-Cog after 1 year

Dependent variable MMSE (12 months)	B	SE	β	p	CI 95%	
					LL	UL
Legumes pattern	0.235	0.095	0.218	0.014	0.04	0.42
Excluded variables: age, education level, SBP						

Dependent variable ADAS-Cog (12 months)	B	SE	β	p	CI 95%	
					LL	UL
Age	0.290	0.106	0.222	0.01	0.07	0.054
Education level	−0.197	0.084	−0.195	0.027	−0.35	−0.022
Legumes pattern	−0.106	0.377	0.246	0.006	−1.79	−0.304
Excluded variables: waist circumferences, glucose						

*CI* confidence interval, *LL* lower limit, *UL* upper limit

**Table 5:** Logistic regression analysis—factors associated with the improved ADAS-Cog

Dependent variable ADAS-Cog improved	B	SE	p	Exp (B)	CI 95%	
					LL	UL
Vegetal protein/polyunsaturated fats	0.584	0.291	0.045	1.79	0.04	0.42
Education level	0.122	0.054	0.023	1.13	1.01	1.25

Excluded variables: SBP, DBP, WC

*CI* confidence interval, *LL* lower limit, *UL* upper limit

## Discussion

In this study we analyse the consumption of foods among elderly Italian individuals using a combination of the dietary intake data, assessed by a 24-h recall and a seven-day diet record, and the PCA to obtain, for the first time, food and nutrient patterns.

In our investigation, four food patterns and four nutrient patterns were identified. However, only two food patterns (legumes and animal fats/margarines patterns) and two nutrient patterns (plant protein/polyunsaturated fats and fats patterns) were associated with the cognitive decline tests.

Furthermore, we found a positive association between a high legumes pattern and the improved MMSE after an interval of 1 year, and an inverse association with basal and 1 year ADAS-cog test. Finally, we found that a dietary pattern that includes plant proteins and polyunsaturated fats is independently associated with an improvement on ADAS-cog after 1 year.

The choice of different foods in elderly have a relevant impact on health. Our results would support the benefit to choose a diet including legumes and plant-based proteins in elderly for preventing cognitive decline. Several previous studies have already demonstrated the beneficial effect of increased MeDi adherence or of individual components of the MeDi on cognitive function, which also belong to other food patterns [1, 6, 7], but there are also negative findings [8]. In addition, no studies have yet found the specific association between a high legumes and high plant-based proteins patterns assessed by PCA with cognitive performance assessed by ADAS-cog.

Several research have provided evidence of diet-induced changes in cerebral aminoacids and, consequently, in neurotransmitters [29–33]. In particular, it has been suggested that tryptophan from animal sources seems less available to synthesized neurotransmitters than those from plant sources, due to a stronger competition with other amino acids [34]. In addition, a relationship between an altered tryptophan metabolism and a high intake of red meat has been identified [35] and the inverse association between processed meat consumption and AD biomarkers has already been established in older individuals [36, 37]. Nevertheless our study was not designed to investigate the mechanisms underlying the association between legumes and cognitive function, all these studies alongside our results, may suggest a greater effect of plant-based proteins on cognitive performance than that of animal protein. Our study also suggests that a dietary pattern including polyunsatured fats may have a positive action on cognitive functions. Thus, in line with other authors [38, 39] we assume that plant-derived foods may prevent cognitive decline. Unlike other studies [38, 39], our investigation highlight the role of both legumes and plant proteins as independent predictors of cognitive performance in the elderly.

Alternatively, other mechanisms could be assumed. Legumes and vegetal oils could improve insulin sensitivity [40], which could, in turn, influence cognitive function. It has been demonstrated that an oral aminoacids mixture significantly improves insulin sensitivity in elderly subjects with sarcopenia or type 2 diabetes, probably by an up-regulation of the insulin-receptor synthesis and its autophosphorylation [41, 42]. Insulin activity could, therefore, be enhanced by legumes and vegetal oils consumption.

However, independently from the underlying mechanisms, our study can be considered relevant as it may reveal a new approach to preventing of the cognitive decline. Our participants in the high legumes pattern consumed more than three serving of legumes per week. Thus, our study should encourage the elderly to increase their weekly legumes intake. Our results are plausible as confirmed by other investigations on this topic. Epidemiologic studies suggest a positive association between flavonoid intake and better cognitive performance in elderly [4]. It has been reported that the cluster “high fish-fruit-vegetable” was associated with a better cognitive function, whereas cluster “moderate ready meals” was associated with cognitive decline of home-living older adults [43]. There is evidence that a nut consumption for 6 months can have positive effects on some cognitive functions of older adults with mild cognitive impairment [44].

Food choices as measured by the combination of a 24-h recall and a seven-day diet record using a specific nutritional software and the PCA were shown to be valid and reproducible [22, 24, 28]. These methods seems to be able to identify individuals with specific preferences but we cannot assess whether there are specific individual characteristics that determine these individual food preferences.

In our investigation we enrolled individuals with a slightly reduced MMSE score (mean 24 ± 1). Nevertheless numerous studies have suggested that the MMSE could differentiate individuals with mild cognitive impairment from those who are normal or have dementia [11, 12], other investigations have suggested that the MMSE lacks the diagnostic accuracy to differentiate between these categories [45]. Consequently, we cannot classify our population neither as with mild cognitive impairment nor as cognitively normal. Aside from that classification, with this investigation we simply generate a new hypothesis for future research and highlight the possible key role of plant proteins in the brain, which remains to be elucidated.

In addition, in our longitudinal observation, only the ADAS-Cog was the main outcome measure. This is not a novelty. In a study performed in elderly persons with normal cognition, the ADAS-Cog was already used as the only test to demonstrate the change in cognitive function overtime [10]. This investigation showed that it is possible to predict decline in cognitively normal individuals [10]. Furthermore, there is consensus that ADAS-cog is a commonly used objective measure of cognitive change in the follow-up evaluation of cognitively impaired individuals [46] and it is able to assess the clinical effects of treatment overtime [18, 47]. Conversely, there is some uncertainty regarding MMSE’s ability to document clinically important change in individual patients over time [48].

In our study, we demonstrated a significant change on ADAS-cog test after 1 year (from 15.4 ± 5 to 13.1 ± 5, p < 0.001). We categorised the population according to the improvement on ADAS-cog after 1 years (i.e. any score reduction; Table 5). At present, a cut-off point on the ADAS cog that accurately classifies patients in respect of their clinical response is not universally accepted, especially with dietary intervention. However, caution must be exercised when interpreting our results on the basis of ADAS-cog change.

In this investigation some weaknesses must be pointed out. Probably MMSE and ADAS-cog may be not ideal for healthy individuals, but these results may suggest interesting hypothesis for future research. Unfortunately, we did not assess the apolipoprotein-E genotype (APOε), however this datum is not necessary or itself sufficient for the development of AD [49].

The participants in this investigation were volunteers since it is very difficult to recruit a random sample of elderly person, as in other studies [18, 45, 50].

Finally, we excluded all subjects taking dietary supplements and psychotropic drugs as ascertained from their medical history and physical examination. However, one could argue that the validity and reliability of self-reports of substance use in elderly is questionable. Furthermore, it cannot be excluded that the medications used affect insulin-resistance and, in turn, cognitive performances.

## Conclusion

To our knowledge, this is the first time that an association of both the legumes and plant proteins with the cognitive performance has been found in elderly individuals using PCA to obtain food and nutrient patterns. Additional studies are required to assess if the change in the ADAS-cog test obtained in this population after one year would result in a reduced AD risk.

## Abbreviations

BMI: body mass index
WC: waist circumference
HC: hip circumference
SBP: systolic blood pressure
DBP: diastolic blood pressure
MMSE: Mini Mental State Examination
ADAS-Cog: Alzheimer’s disease assessment scale-cognitive sub-scale
PCA: Principal Components Analysis
